# Spatial Gradients of E-Cadherin and Fibronectin in TGF-β1-Treated Epithelial Colonies Are Independent of Fibronectin Fibril Assembly

**DOI:** 10.3390/ijms24076679

**Published:** 2023-04-03

**Authors:** Lauren A. Griggs, Christopher A. Lemmon

**Affiliations:** 1 Center for Engineering Outreach and Inclusion, Pennsylvania State University, University Park, PA 16802, USA; 2 Department of Biomedical Engineering, Virginia Commonwealth University, Richmond, VA 23284, USA

**Keywords:** fibronectin, epithelial mesenchymal transition, micropatterning, TGF-β1

## Abstract

Epithelial to Mesenchymal Transition (EMT) is a dynamic, morphogenetic process characterized by a phenotypic shift in epithelial cells towards a motile and often invasive mesenchymal phenotype. We have previously demonstrated that EMT is associated with an increase in assembly of the extracellular matrix protein fibronectin (FN) into insoluble, viscoelastic fibrils. We have also demonstrated that Transforming Growth Factor-β1 (TGF-β1) localizes to FN fibrils, and disruption of FN assembly or disruption of TGF-β1 localization to FN fibrils attenuates EMT. Previous studies have shown that TGF-β1 induces spatial gradients of EMT in mammary epithelial cells cultured on FN islands, with cells at free edges of the island preferentially undergoing EMT. In the current work, we sought to investigate: (a) whether FN fibril assembly is also spatially patterned in response to TGF-β1, and (b) what effects FN fibril inhibition has on spatial gradients of E-Cadherin and FN fibrillogenesis. We demonstrate that mammary epithelial cells cultured on square micropatterns have fewer E-Cadherin-containing adherens junctions and assemble more FN fibrils at the periphery of the micropattern in response to increasing TGF-β1 concentration, indicating that TGF-β1 induces a spatial gradient of both E-Cadherin and FN fibrils. Inhibition of FN fibril assembly globally diminished E-Cadherin-containing adherens junctions and FN fibrillogenesis, but did not eliminate the spatial gradient of either. This suggests that global inhibition of FN reduces the degree of both FN fibrillogenesis and E-Cadherin-containing adherens junctions, but does not eliminate the spatial gradient of either, suggesting that spatial gradients of EMT and FN fibrillogenesis are influenced by additional factors.

## 1. Introduction

Epithelial-Mesenchymal Transition (EMT) is a transdifferentiation process in which epithelial cells transition into a mesenchymal phenotype. This process is associated with a loss of apicobasal polarity and collective migration (reviewed in [1,2]), as well as an increase in extracellular matrix (ECM) assembly [3,4]. EMT plays a critical role in wound healing and development, but has also been implicated as a prominent mechanism in epithelial-derived tumors [5,6,7] and fibrotic diseases (reviewed in [8]).

EMT can be induced in epithelial monolayers by exposure to the inflammatory cytokine TGF-β1. Previous studies have demonstrated that TGF-β1 induces EMT in a spatially-dependent pattern in mammary epithelial cells cultured on FN islands, with EMT primarily occurring at the edges of cell islands and not within the center [9,10]. This spatial gradient has been attributed to high intracellular tension at island edges [9,11]; inhibition of actomyosin contractility eliminated this spatial gradient.

**Figure 1:**
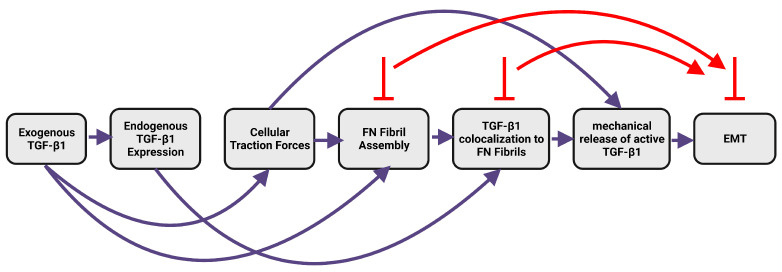
The relationship between TGF-β1, fibronectin fibrillogenesis, and Epithelial-Mesenchymal Transition. Prior work has shown that TGF-β1 induces upregulation of contractile forces [26], which in turn upregulate fibronectin fibrillogenesis [3,17]. Endogenous TGF-β1 localizes to fibronectin fibrils [27] to promote EMT. Inhibition of either fibronectin fibrillogenesis or TGF-β1 to fibronectin fibrils inhibits EMT [4].

The ECM plays a key role in regulating both chemical and mechanical signaling within the tumor microenvironment, and has been implicated in EMT [3,12]. ECM stiffness has been shown to regulate a switch in TGF-β signaling, in which TGF-β1 induces apoptosis on soft ECM and induces EMT on stiff ECM [13]. Assembly of the ECM protein fibronectin (FN) into insoluble fibrils is upregulated during EMT and has also been implicated as a key step in TGF-β1-induced EMT [4,14]. FN fibril formation is a complex process: integrins on the cell surface bind to soluble FN, and stretch it via actomyosin contractility to expose cryptic FN-FN binding sites [15,16,17,18]; exposure of these sites allows for the binding of a second FN molecule, which creates another integrin binding site. This process continues until a viscoelastic, insoluble fibril has formed. FN fibrils alter both the mechanics of the local environment [19,20,21] and also play a key role in growth factor signaling events [4,22]: a fragment of the FN protein, which is only exposed in FN fibrils, can bind and tether upwards of 40 different growth factors and cytokines [23]. Of particular relevance to the current work is the binding of TGF-β1 to FN fibrils, which occurs via binding of the large latent complex of TGF-β1, which includes active TGF-β1, its Latency Associated Peptide (LAP), and the Latent TGF-β1 Binding Protein (LTBP1) [24,25]. Previous work has demonstrated that exogenous TGF-β1 drives an increase in endogenous TGF-β1 expression as well as increased FN fibrillogenesis (Figure 1, [4]). Inhibition of either FN fibrillogenesis or localization of TGF-β1 to FN fibrils inhibits EMT [4].

The ECM additionally plays a key role in establishing spatial gradients in a variety of biological venues, including during embryonic development and tissue regeneration. ECM stiffness has been shown to control intercellular stress, which in turn regulates processes involved in organ branching [28]. In addition, geometric cues provided by the ECM have been shown to guide cellular protrusions, resulting in regulation of cell shape and migration [29]. Tracks of FN fibrils have been shown to guide cell spreading and directional persistence during migration [30].

Taken together, we hypothesized that assembly of FN fibrils may play a key role in establishing the spatial gradients of EMT within tissues. We investigated this by growing micropatterned islands of epithelial cells and probing the effects of FN fibril inhibition on spatial patterns of EMT within the islands.

## 2. Results

### 2.1. FN Fibril Assembly Is Spatially Patterned in Micropatterned Epithelial Colonies

**Figure 2:**
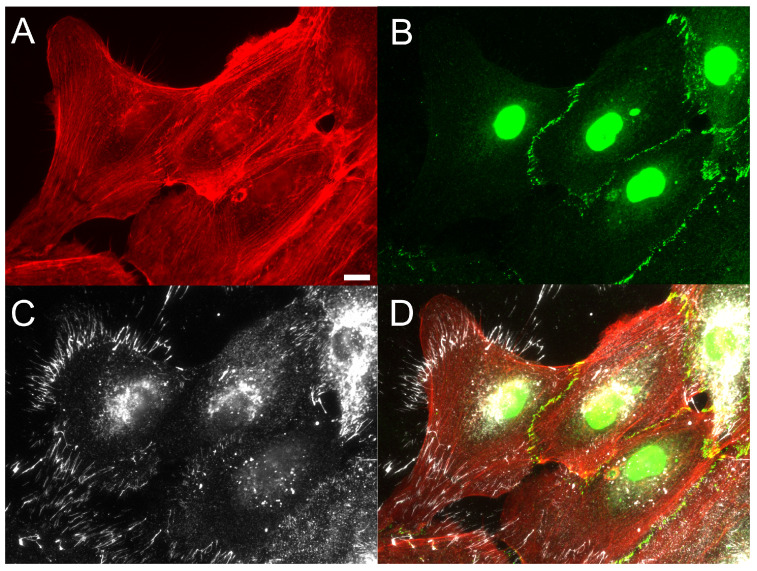
Fibronectin fibrils preferentially occur at cell voids in epithelial monolayers. Studies of incomplete epithelial cell monolayers indicated that FN fibril assembly preferentially occurred at free edges of the monolayer in the presence of 2 ng/mL TGF-β1. Immunofluorescence images of (**A**) F-actin (red), (**B**) E-cadherin (green), and (**C**) FN (white) are shown ((**D**) composite image). Scale bar 10 μm.

**Figure 3:**
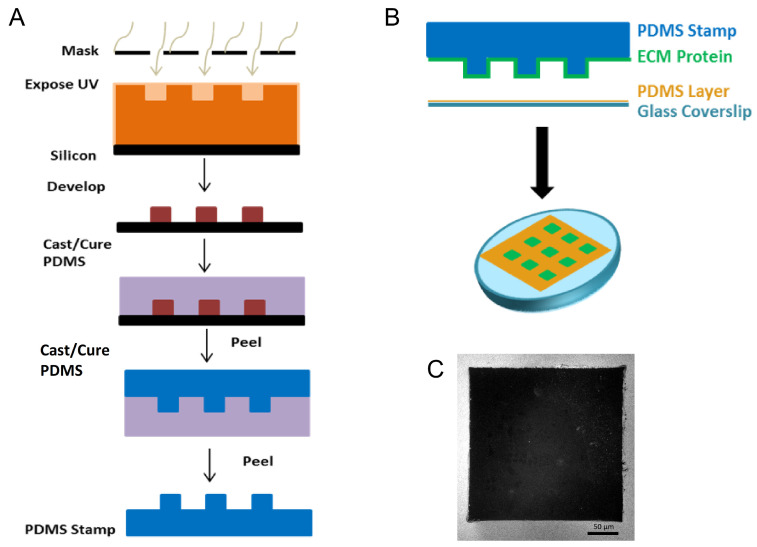
Fabrication of substrates to generate repeatable epithelial cell colonies. (**A**) To study this in a controlled environment, we utilized photolithography to generate stamps that consisted of 250 μm × 250 μm raised squares. (**B**) The process for microcontact printing an array of 250 μm × 250 μm squares onto polydimethyl-siloxane (PDMS)-coated glass. (**C**) Representative immunofluorescence image of fluorescently labeled BSA (white) showing the inverse of a 250 μm × 250 μm square. Scale bar 50 μm.

**Figure 4:**
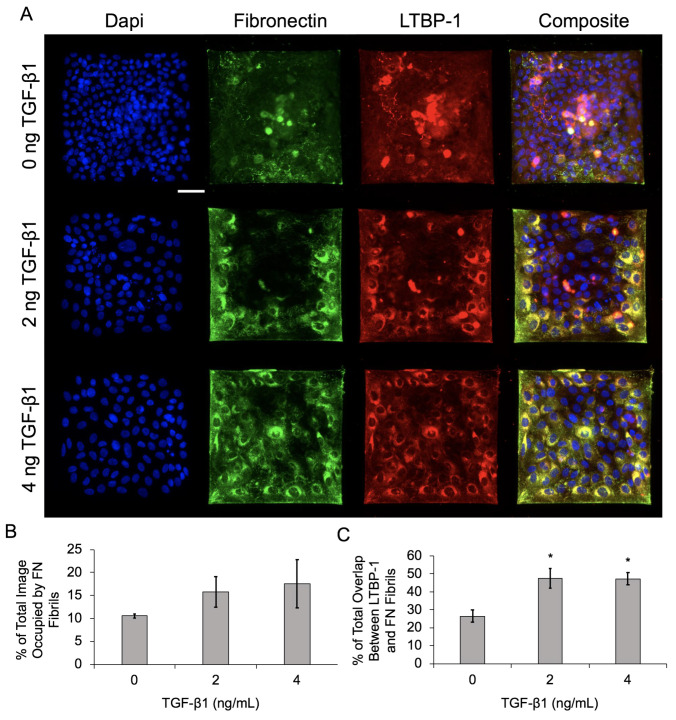
Increasing TGF-β1 concentration upregulates FN fibril assembly and LTBP-1 colocalization with FN fibrils in MCF10As on laminin islands. (**A**) Immunofluorescence images of MCF10As after 48 h of incubation with increasing concentrations of TGF-β1. Antibody staining for nuclei (blue), FN (green), and LTBP-1 (red). Composite displays colocalization of FN and LTBP-1 in yellow. Scale bar 50 µm. (**B**) Percentage of image occupied by FN fibrils. (**C**) Quantification of colocalized LTBP-1 on FN fibrils. N = 3 experimental replicates, *n* > 10 images per experiment for each condition. * *p* ≤ 0.05 significantly different from 0 ng/mL TGF-β1. *p*-values based on Bonferroni’s post-hoc multiple comparisons test following one-way ANOVA.

Previous work from our group demonstrated that TGF-β1 induces assembly of FN fibrils in epithelial monolayers [4]. We have observed that cells at the free edges of these monolayers preferentially assemble FN fibrils, and that these cells exhibit pronounced morphological changes consistent with EMT (Figure 2). Given that others have observed preferential EMT at free edges of colonies [9], we hypothesized that FN fibril assembly at the free edge may be responsible for spatial gradients of EMT. To investigate this, we used photolithography and microcontact printing to engineer 250 μm × 250 μm laminin islands on poly-dimethyl siloxane (PDMS) surfaces (Figure 3). MCF10A human mammary epithelial cells were then cultured on these laminin islands to produce consistent epithelial colonies with identical dimensions. MCF10As have previously been shown to undergo EMT in response to TGF-β1 [4,13] and are representative of non-transformed mammary epithelial cells. Patterned islands contained roughly 100 cells and were of sufficient size to observe spatial gradients across the colony. Cells were incubated on the islands for 18 h to allow colonies to reach confluence; cells were then serum and growth factor starved for 2 h before being incubated for 48 h in the presence of 0, 2 or 4 ng/mL TGF-β1. Colonies were immunofluorescently labeled for nuclei, FN, and Latent-TGF-β-binding protein-1 (LTBP-1), which is secreted as part of the TGF-β1 complex and which localizes to FN fibrils [31]. Representative images of epithelial colonies cultured in 0, 2 or 4 ng/mL TGF-β1 are shown in Figure 4A. Note that nucleus images indicate a uniform fluoresence intensity across the colony, indicating that this pattern of increased intensity at the island periphery is not an artifact of staining.

Results indicate that FN fibril assembly increased with an increasing concentration of TGF-β1 (Figure 4B), which is consistent with our previous data on epithelial monolayers [4]. Localization of LTBP-1 and FN also increased with increasing TGF-β1, again consistent with previous studies. Qualitatively, there is an observable spatial gradient of FN fibril formation and LTBP-1 localization in Figure 4A, with increases in both at the periphery of colonies.

**Figure 5:**
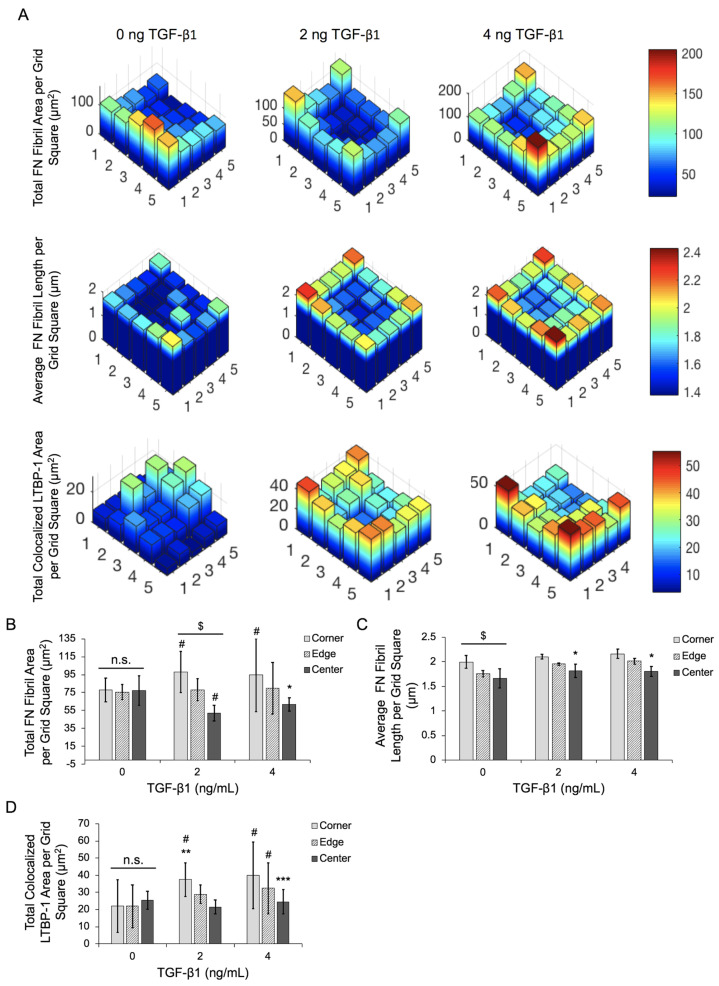
Increasing TGF-β1 concentration induces spatial gradients of FN fibril area, FN fibril length, and LTBP-1 colocalization with FN fibrils in MCF10A square islands. (**A**) Data from cell islands was partitioned into a 5 × 5 grid. Representative frequency maps of total FN fibril area per grid square, average FN fibril length per grid square and total colocalized LTBP-1 per grid square are shown. Grid regions were separated into corner, edge, and center regions for quantification of spatial gradients. (**B**) Total FN fibril area per grid square based on TGF-β1 concentration and square location. (**C**) Average FN fibril length per grid square based on TGF-β1 concentration and square location. (**D**) Total LTBP-1/FN colocalization area per grid square based on TGF-β1 concentration and square location. N = 3 experimental replicates, n > 10 islands per experiment for each condition. n.s., not significant, * *p* ≤ 0.05 significantly different from both edge and corner within given concentration, ** *p* ≤ 0.05 significantly different from both edge and center within given concentration, *** *p* ≤ 0.05 significantly different from only corner within given concentration, ${}^{\$}$ *p* ≤ 0.05 significantly different from all other locations within given concentration, ${}^{\#}$ *p* ≤ 0.05 significantly different from corresponding location in 0 ng/mL TGF-β1. *p*-values based on Bonferroni’s post-hoc multiple comparisons test following Two-way ANOVA.

To quantify this spatial gradient, immunofluorescence images were binned into 5 × 5 grids, and analysis was performed separately in each binned region. Binned data was averaged across all colonies in the experiment, and frequency maps were generated, showcasing the average value in each binned region (Figure 5A). Further analysis was performed by combining these bins into three categories: corner bins (four bins per colony), non-corner edge bins (12 per colony), and center bins (nine per colony). The average FN fibril area (Figure 5B), average FN fibril length (Figure 5C), and colocalized area of LTBP-1 and FN (Figure 5D) were calculated as functions of both spatial location (corner, edge, or center) and TGF-β1 dose. Data show that in the absence of TGF-β1, there is no spatial variation in FN fibril area or LTBP-1 colocalization to FN. FN fibril length exhibited some spatial variation, with longer fibrils in the corners of colonies. As TGF-β1 concentration increased, spatial variation in fibril area, fibril length, and LTBP-1 colocalization increased, with corners of the colonies exhibiting the largest and longest fibrils and the most LTBP-1 colocalization, edge regions showing less, and center regions showing the smallest fibrils with the least colocalization of LTBP-1.

### 2.2. E-Cadherin-Containing Adherens Junctions Exhibit Spatial Gradients in Epithelial Colonies

**Figure 6:**
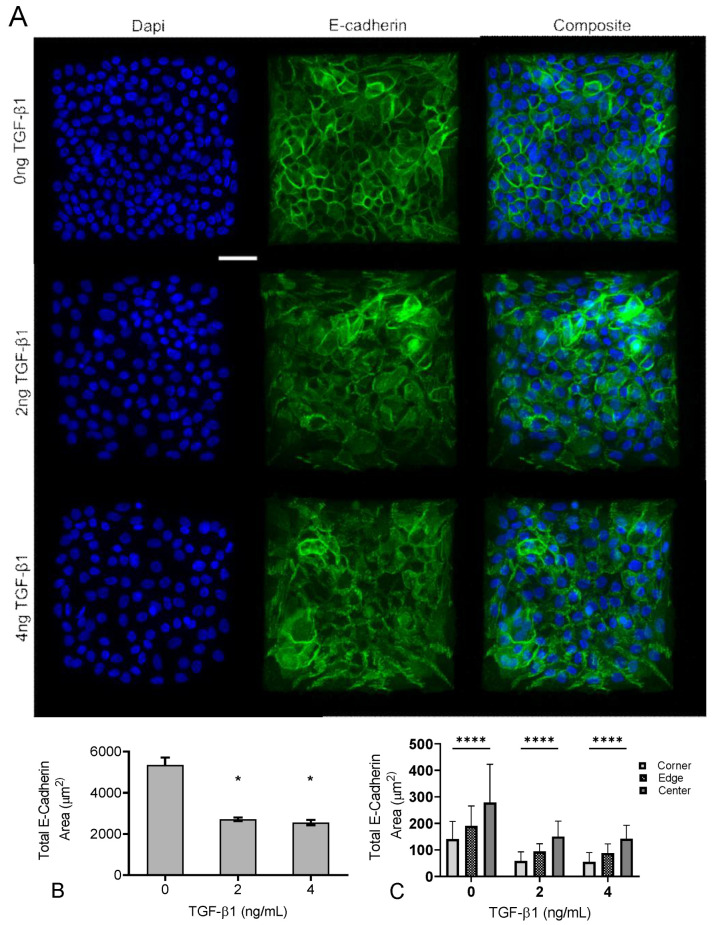
Increasing TGF-β1 concentration disrupts adherens junctions in MCF10A square islands. (**A**) Representative immunofluorescence images of MCF10A cells cultured for 48 h with increasing concentrations of TGF-β1. Ab staining for nuclei (blue), and E-cadherin (green). Scale bar 50 mm. (**B**) Total E-Cadherin area in immunofluorescence images. (**C**) Scale bar 50 μm. * *p* ≤ 0.05 significantly different from control, **** *p* ≤ 0.05 significantly different from both edge and center within given concentration. *p*-values based on Bonferroni’s post-hoc multiple comparisons test following Two-way ANOVA.

We next examined adherens junctions in the epithelial colonies in response to increasing concentrations of TGF-β1. A prominent endpoint of EMT signaling is the loss of E-Cadherin from adherens junctions [32]. To quantify both the degree of E-Cadherin loss and the spatial gradient of E-Cadherin loss, we immunofluorescently labeled cells for E-Cadherin and quantified the junctional area containing E-Cadherin. Representative images are shown in Figure 6A, and quantified E-Cadherin area is shown in Figure 6B. Data indicate that as TGF-β1 concentration increases, there is a reduction in E-Cadherin area. We then quantified spatial localization of E-cadherin-containing adherens junctions, again dividing the islands into a 5 × 5 bin and quantifying E-Cadherin area in each region (corner, edge, and center) (Figure 6C). Results indicate a spatial localization of E-Cadherin-containing junctions, with smaller E-Cadherin area in the corners, relative to the edge and center of the cell. While TGF-β1 decreased E-Cadherin area, spatial localization of E-Cadherin was observed across all doses of TGF-β1. These data demonstrate that E-Cadherin-containing junctions are spatially patterned, consistent with previously published work [9], but suggest that this gradient occurs independent of TGF-β1.

### 2.3. Inhibition of FN Fibril Assembly Decreases Overall Assembly but Retains Spatial Gradients

Having established that TGF-β1 drives an increase in spatial gradients of both FN fibrils and LTBP-1 colocalization to FN fibrils, and that this pattern corresponded with spatial gradients of EMT, we next sought to investigate how these spatial patterns would be affected by inhibition of FN fibril formation. We sought to investigate two possible mechanisms: (1) TGF-β1 induces spatial gradients of FN fibril formation, and this in turn induces spatial gradients of EMT in the regions where FN assembly is more prevalent; or (2) TGF-β1-induced assembly of FN fibrils create a permissive environment for EMT, and the geometry of the colony then dictates where EMT (and FN fibril formation) preferentially happens. In other words, do spatial gradients of FN induce spatial gradients of EMT, or does the presence of any FN, regardless of spatial gradients, induce EMT, with spatial gradients of EMT driven by other inputs (such as the geometry of the island)?

**Figure 7:**
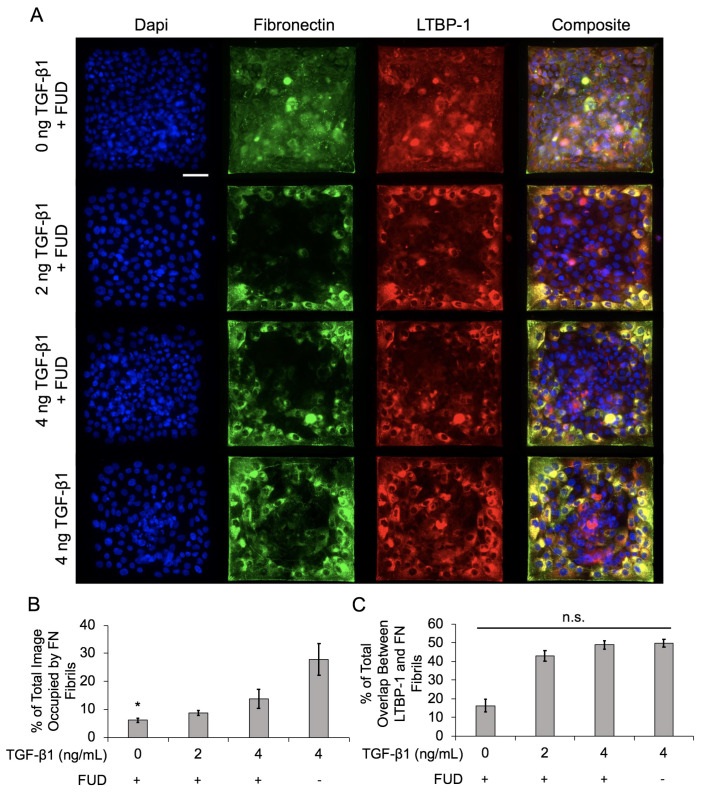
Inhibition of FN fibrillogenesis decreases overall FN assembly but does not affect LTBP-1 colocalization with FN fibrils in MCF10A square islands with increasing TGF-β1 concentration. (**A**) Immunofluorescence images of MCF10As after 48 h of treatment with FUD and increasing concentrations of TGF-β1. Ab staining for nuclei (blue), FN (green), and LTBP-1 (red). Composite displays colocalization of FN and LTBP-1 in yellow. Scale bar 50 mm. (**B**) Quantification of percentage of image occupied by FN fibrils. N = 3 for each condition. (**C**) Quantification of colocalized LTBP-1 on FN fibrils. N = 3 for each condition. n.s., not significant, * *p* ≤ 0.05 significantly different from positive control only, 4 ng/mL TGF-β1. *p*-values based on Bonferroni’s post-hoc multiple comparisons test following one-way ANOVA.

To address this, we treated epithelial cell islands with the Functional Upstream Domain (FUD) of the S. Pyogenes protein F1 Adhesin. Previous studies have demonstrated that this 49-amino acid fragment is a potent inhibitor of FN fibril formation, but does not affect FN secretion or integrin binding to FN [33]. Cells were treated with 125 nM FUD, which has previously been shown to be an effective dose for inhibiting FN fibril assembly [4], along with 0, 2, or 4 ng/mL TGF-β1. Cell islands were treated with 4 ng/mL TGF-β1 alone (without FUD) as a positive control. Representative images of nuclei, FN, and LTBP-1 are shown in Figure 7A. Results indicate that FN fibril formation was significantly reduced relative to the positive control (Figure 7B). Despite the reduced assembly of FN fibrils, we observed a similar degree of colocalization of LTBP-1 to FN fibrils (Figure 7C), suggesting that while there are fewer FN fibrils in the FUD-treated cases, there is still a similar degree of latent TGF-β1 localized to these fibrils.

**Figure 8:**
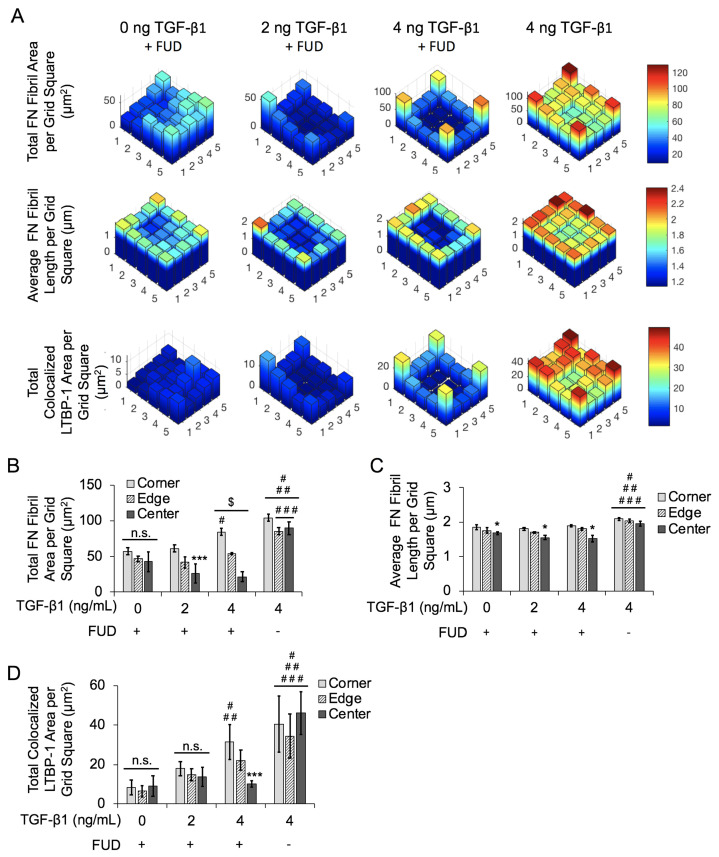
Inhibition of FN fibrillogenesis reduces effects of spatial gradients of FN fibril area, FN fibril length, and LTBP-1 colocalization with FN fibrils in MCF10A square islands with increasing concentrations of TGF-β1. (**A**) Representative frequency maps of total FN fibril area per grid square, average FN fibril length per grid square and total colocalized LTBP-1 per grid square. (**B**) Quantification of total FN fibril area per grid square based on TGF-β1 concentration and square location. N = 3 for each condition. (**C**) Quantification of average FN fibril length per grid square based on TGF-β1 concentration and square location. N = 3 for each condition. (**D**) Quantification of total LTBP-1 colocalized area per grid square based on TGF-β1 concentration and square location. N = 3 for each condition. n.s., not significant, * *p* ≤ 0.05 significantly different from both edge and corner within given concentration, *** *p* ≤ 0.05 significantly different from only corner within given concentration, ^$^ *p* ≤ 0.05 significantly different from all other locations within given concentration, ^#^ *p* ≤ 0.05 significantly different from corresponding location in 0ng/mL TGF-β1 only, ^##^ *p* ≤ 0.05 significantly different from corresponding location in 2 ng/mL TGF-β1 only, ^###^ *p* ≤ 0.05 significantly different from corresponding location in 4 ng/mL TGF-β1 only. *p*-values based on Bonferroni’s post-hoc multiple comparisons test following two-way ANOVA.

We next examined the spatial gradients of FN fibrils as before, dividing the islands into 5 × 5 bins and averaging values in each bin. We again collated data for the four corner bins, the 12 edge bins, and and the nine center bins (Figure 8). The average value for each bin is shown in Figure 8A for total FN fibril area, FN fibril length, and LTBP-1 colocalization to FN fibrils. Results indicate that even though the overall degree of FN fibril formation is reduced relative to the positive control, TGF-β1 still induces a spatial gradient of FN, with more and larger fibrils in the corners and edges of the square. LTBP-1 colocalization is also greater in the corners and edges of the square relative to the center. These data indicate that while FUD reduces the overall degree of FN fibrillogenesis, a spatial gradient of FN fibril formation is still maintained.

### 2.4. Inhibition of FN Fibril Assembly Globally Inhibits E-Cadherin, Despite Persistence of FN Spatial Gradient

**Figure 9:**
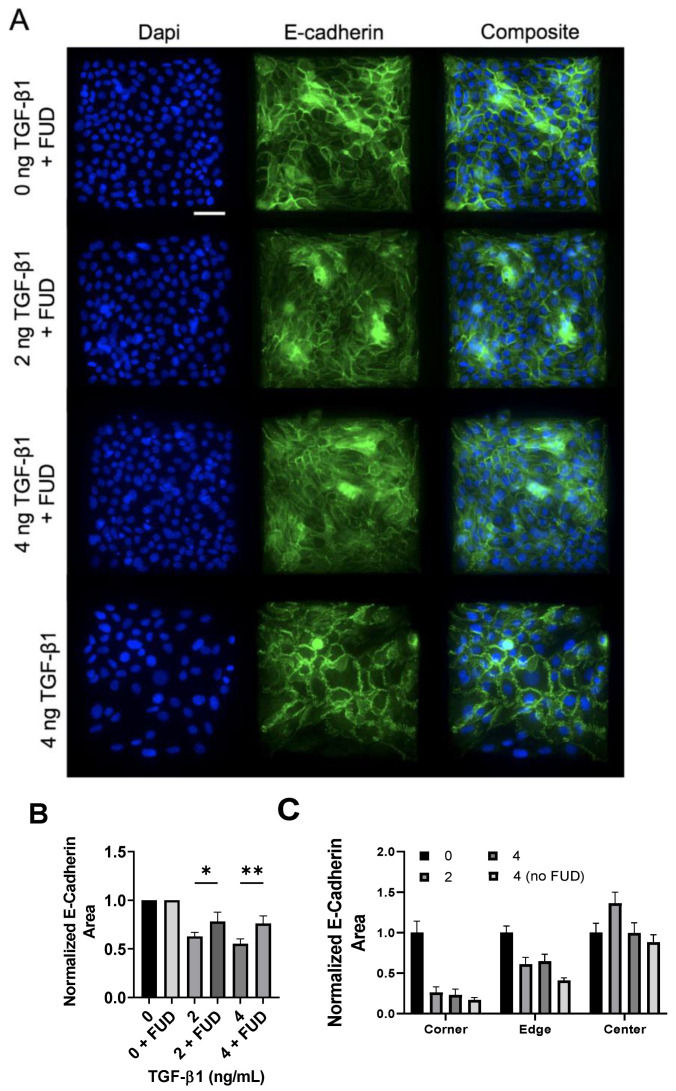
Inhibition of FN fibrillogenesis partially blocks TGF-β1-induced E-Cadherin loss, but maintains spatial gradients of E-Cadherin. (**A**) Immunofluorescence images of MCF10As after 48 h of treatment with FUD and increasing concentrations of TGF-β1. Ab staining for nuclei (blue) and E-Cadherin (green). Scale bar 50 µm. (**B**) Quantification of E-Cadherin area. E-Cadherin area per image was normalized by the average E-Cadherin area in the negative control condition (0 ng TGF-β1). N = 3 for each condition. (**C**) Quantification of spatial gradients of E-Cadherin area. E-Cadherin area was normalized by the average E-Cadherin area in the corner, edge and center regions, respectively, from the negative control condition (0 ng TGF-β1). N = 3 for each condition. * *p* ≤ 0.05, ** *p* ≤ 0.01. *p*-values based on Bonferroni’s post-hoc multiple comparisons test following one-way ANOVA.

To assess the effects of FN fibril inhibition on E-Cadherin, we again treated cells with 125 nM FUD along with 0, 2, or 4 ng/mL TGF-β1. Cell islands were again treated with 4 ng/mL TGF-β1 alone (without FUD) as a positive control. Representative images are shown in Figure 9 and show that TGF-β1-dependent decreases in E-Cadherin are significantly lessened in the presence of FUD (Figure 9B).

We then quantified spatial localization of E-Cadherin-containing junctions in the presence of the FN assembly inhibitor. As before, images were binned into a 5 × 5 grid, and values in each bin were averaged. These bins were then combined into corner, edge, and center bins. Results indicate that spatial localization is again maintained, even in the presence of FUD. This suggests that despite the inhibition of FN fibril assembly, spatial patterns of E-Cadherin persist.

## 3. Discussion

It has previously been established that spatial gradients of EMT markers occur in epithelial colonies [9,10,11]. It is known that TGF-β1 stimulates these gradients; however, much is left to be understood about spatial gradients in the context of the ECM and downstream morphological events. Here, we characterized TGF-β1-induced spatial gradients of FN fibrils, LTBP-1 colocalization with FN fibrils, and E-Cadherin content in adherens junctions, which is a prominent marker of EMT. We showed that with increasing TGF-β1 concentration, FN fibrils were preferentially assembled at corners and edges of square epithelial islands. LTBP-1 colocalization with FN fibrils was also spatially concentrated at corners and edges in epithelial islands with stimulation from TGF-β1. We also showed that EMT is spatially patterned on these islands, with fewer E-cadherin-containing adherens junctions at the corners and edges of squares relative to the center. To ascertain the importance of FN assembly and/or FN spatial gradients on spatial gradients of EMT, we inhibited FN fibril formation with a bacterial peptide that has previously been shown to be a potent inhibitor of FN fibril assembly. Our results indicated that while overall FN fibril assembly was reduced, the spatial gradients of FN fibrils persisted. Examining E-Cadherin, our results indicated that the inhibition of FN fibril formation blocked the overall amount of E-Cadherin within junctions, but did not disrupt spatial gradients of E-Cadherin. These results suggest that the overall amount of FN fibril assembly, and not the spatial gradients of FN, dictates both the degree of EMT as well as the spatial gradients of EMT.

Previous work from our lab has demonstrated that the presence of FN fibrils creates a permissive environment for cells to generate larger contractile forces. Previous studies have also shown that (a) larger forces can drive EMT [9,34] and (b) islands of cells generate larger forces at the periphery of the island [28]. As such, we envision that our data points to the following mechanism: soluble TGF-β1 drives an increase in contractility, which facilitates FN assembly. The presence of FN fibrils facilitate larger contractile forces, and this positive feedback loop establishes both a spatial gradient of FN fibrils and a spatial gradient of traction forces. It is the latter that then facilitates the spatial gradients of EMT in the island. When FN fibril formation is inhibited, there is a global reduction in both FN fibril formation and traction forces, which is still spatially patterned, but which drops traction forces below a threshold that can induce EMT. This thus blocks both the overall degree of EMT but not the spatial gradients of EMT. Future work will probe this hypothesis by implementing assays to quantify traction forces in the patterned islands as we have previously shown [26].

## 4. Materials and Methods

### 4.1. Cell Culture and Reagents

All cells were cultured in a humidified atmosphere at 37 °C with 5% CO_2_. Human MCF10A mammary epithelial cells were obtained from the National Cancer Institute Physical Sciences in Oncology Bioresource Core Facility, in conjunction with American Type Culture Collection (Manassas, VA, USA). MCF10As were maintained under standard culture conditions in DMEM/F-12 HEPES (Life Technologies, Carlsbad, CA, USA), supplemented with the following supplements (Sigma Aldrich, St. Louis, MO, USA): 5% horse serum, 0.05% hydrocortisone, 0.01% cholera toxin, 0.1% insulin, 0.02% EGF and 1% antibiotics. Purified recombinant active TGF-β1 was purchased from Sigma Aldrich (St. Louis, MO, USA). Immunofluorescence imaging was conducted using primary antibodies: Ms anti-Hu E-cadherin (HECD-1, Abcam, Cambridge, UK), Rb anti-Hu FN (Abcam, Cambridge, UK), Ms anti-Hu LTBP-1 (R & D Systems, Minneapolis, MN, USA), Ms anti-Ms N-cadherin (BD Biosciences, San Jose, CA, USA). F-actin images were acquired by labeling cells with AlexaFluor555 Phalloidin (Life Technologies, Carlsbad, CA, USA). Nuclei images were acquired by labeling cells with NucBlue Fixed Cell Stain ReadyProbes reagent (Invitrogen, Carlsbad, CA, USA).

### 4.2. Generation of Microcontact Printed Surfaces

Uniform square islands were generated as previously described [35,36,37]. Briefly, a negative mold of an array of 250 µm × 250 µm squares was fabricated through photolithography on a silicon wafer (Figure 3). A replica-mold of polydimethylsiloxane (PDMS) raised stamps was coated with laminin (LN). The stamp was then placed into contact with UV-treated, PDMS coated, glass coverslips to facilitate covalent bonding. Coverslips were fabricated through spin coating of a thin layer (>10 µm) of Sylgard 184 PDMS (1:10 *w:w* curing agent to base) onto the coverslips and baking overnight at 60 °C. After stamping, coverslips were treated with fluorescently labeled BSA to label the region outside of the square pattern and 1% Pluronics F-127 to prevent cells from adhering outside of the square patterns. LN was used to coat stamps to prevent acquisition of confounding results from FN interactions.

### 4.3. Expression and Purification of FUD

cDNA for FUD was inserted into a bacterial expression vector that contains a C-terminal polyhistidine tag and maltose binding protein (MBP), both of which facilitate protein purification. cDNA was obtained from Dr. Harold Erickson, Duke University Medical Center, with permission from Dr. Deane Mosher at the University of Wisconsin. FUD expression, purification and determination of recombinant protein concentration has been previously described [9]. FN fibril formation was inhibited by addition of 125 nM FUD in MCF10As.

### 4.4. Experimental Design and Immunofluorescence Imaging

First, 0.5 × $10^{6}$ MCF10As were cultured on 250 µm × 250 µm microcontact printed square islands coated with LN. Cells were grown overnight, then EGF/serum starved, at which point the islands reached between 50 and 70% confluence. In experiments with FN fibril inhibition, 125 nM FUD was introduced into culture two h after EGF/serum starvation and 1 h prior to TGF-β1 addition. Cells were incubated with TGF-β1 (0, 2, 4 ng/mL) and cultured for an additional 48 h. Cells were then permeabilized with 0.5% Triton in 4% paraformaldehyde for 2 min, then incubated in 4% paraformaldehyde for 20 min. Fixed cells were rinsed in 1× phosphate buffered saline (PBS), followed by blocking in 0.1% BSA in PBS and labeling with primary antibody for 30 min at 37 °C. Cells were then blocked again in 0.1% BSA and incubated with the appropriate secondary antibody for 30 min. Images were acquired on a Zeiss AxioObserver Z1 fluorescence microscope using Zen software.

### 4.5. FN Fibril Quantification

To quantify FN fibrillogenesis, immunofluorescence images of FN were analyzed via an image processing algorithm in Matlab that creates a binary mask of each FN image and determines the total area occupied by FN fibrils per image. These values were normalized by the total image size.

### 4.6. Average FN Fibril Length Quantification

To quantify average FN fibril length, immunofluorescence images of FN were analyzed via an image processing algorithm in Matlab that creates a binary mask of each FN image and determines the length of a fibril based on the shortest path along the fibril from end point to end point.

### 4.7. LTBP-1 Colocalization Quantification

To quantify LTBP-1 colocalization with FN fibrils, immunofluorescence images of LTBP-1 and FN were analyzed via an image processing algorithm in Matlab that creates a binary mask of each LTBP-1 image, as well as each FN image, and determines the areas in which LTBP-1 overlays FN fibrils per image.

### 4.8. Spatial Gradient Analysis

To quantify spatial gradients, analysis of island data was separated into corners, edges, and centers via an image processing algorithm in Matlab. Islands were first divided into 5 × 5 grids. Analyses including FN fibril quantification, average FN fibril length, LTBP-1 colocalization, and E-Cadherin area were performed separately in each of the binned regions. These values were then summed across the same bin space in each image in a given condition to create a frequency map. Corner data was defined as the average of the four corner bins, edge data was defined as the average of the 16 outer bins, and center data was defined as the average of the 9 inner bins.

### 4.9. Statistical Analysis

All statistics were determined through either a one- or two-way ANOVA followed by Bonferroni’s post-hoc multiple comparisons test. Statistics were analyzed via GraphPad Prism.

## 5. Conclusions

There is a complex relationship between an epithelial cell and its surrounding matrix, and this relationship dictates the cellular response to EMT-inducing stimuli. EMT is a critical cell process for both physiological events such as wound healing and organogenesis; the process is also often misregulated in pathological settings. In the current work, we demonstrated that FN fibril assembly and localization of TGF-β1 to FN fibrils occurs in a spatial gradient across epithelial colonies, and this spatial gradient correlates with a spatial gradient of E-Cadherin-containing adherens junctions. The disruption of FN fibril assembly inhibited both the global amount of E-Cadherin containing junctions and the number of FN fibrils; however, spatial gradients of both still persisted. This suggests that the establishment of spatial gradients of EMT is independent of fibronectin fibril formation.

## Data Availability

The data presented in this study are available on request from the corresponding author.

## Abbreviations

The following abbreviations are used in this manuscript:

EMT	Epithelial-Mesenchymal Transition
FN	Fibronectin
FUD	Functional Upstream Domain of F1-Adhesin
LAP	Latency Associated Peptide
LN	Laminin
LTBP-1	Latent TGF-β Binding Protein-1
TGF-β1	Transforming Growth Factor β1
